# Drug Delivery Approaches for Managing Overactive Bladder (OAB): A Systematic Review

**DOI:** 10.3390/ph14050409

**Published:** 2021-04-26

**Authors:** Zara Khizer, Amina Sadia, Raman Sharma, Samia Farhaj, Jorabar Singh Nirwan, Pratibha G. Kakadia, Talib Hussain, Abid Mehmood Yousaf, Yasser Shahzad, Barbara R. Conway, Muhammad Usman Ghori

**Affiliations:** 1 Department of Pharmacy, School of Applied Sciences, University of Huddersfield, Huddersfield HD1 3DH, UK; zara.khizer@hud.ac.uk (Z.K.); samia.farhaj@hud.ac.uk (S.F.); jorabar.nirwan@hud.ac.uk (J.S.N.); b.r.conway@hud.ac.uk (B.R.C.); 2 District Headquarter Hospital, Sahiwal 57000, Pakistan; draminasadia@yahoo.com; 3 Parkside Medical Practice, Horton Park Health Centre, Bradford BD7 3EG, UK; raman.sharma7@nhs.net; 4 Behavioral & Social Science, Integrated Behavioral Health Research Institute, San Gabriel, CA 91775, USA; pratibhakakadia@gmail.com; 5 Department of Pharmacy, COMSATS University Islamabad, Lahore Campus, Lahore 45550, Pakistan; t.h.kayani@live.com (T.H.); abid.ucp@hotmail.com (A.M.Y.); y.shahzad@live.com (Y.S.)

**Keywords:** overactive bladder, drug delivery, transdermal drug delivery, pharmacotherapy, oral drug delivery, systematic review

## Abstract

Overactive bladder syndrome (OAB) is characterised by urgency symptoms, with or without urgency incontinence, usually with frequency and nocturia and severely affects the quality of life. This systematic review evaluates the various drug delivery strategies used in practice to manage OAB. Advanced drug delivery strategies alongside traditional strategies were comprehensively analysed and comparatively evaluated. The present review was conducted according to the preferred reporting items for systematic reviews and meta-analyses guidelines. A total of 24 studies reporting the development of novel formulations for the treatment of OAB were considered eligible and were further categorised according to the route of drug administration. The review found that various drug delivery routes (transdermal, intravesicular, oral, vaginal and intramuscular) are used for the administration of drugs for managing OAB, however, the outcomes illustrated the marked potential of transdermal drug delivery route. The findings of the current review are expected to be helpful for pharmaceutical scientists to better comprehend the existing literature and challenges and is anticipated to provide a basis for designing and fabricating novel drug delivery systems to manage OAB.

## 1. Introduction

Overactive bladder (OAB) syndrome is characterised by *“urinary urgency, usually accompanied by frequency and nocturia, with or without urgency urinary incontinence, in the absence of urinary tract infection (UTI) or other obvious pathology”* [1]. According to a population-based survey carried out in the United Kingdom, Canada, Sweden, Italy and Germany, the overall prevalence of OAB was estimated to be 11.8% for both men and women and was found to increase with age [2]. However, this estimate may be an under-representation of the true prevalence of OAB as many patients may be reluctant and unwilling to discuss their condition with family members or healthcare providers, as observed in a population-based survey in which fewer than 50% of respondents with probable OAB had discussed their condition with their healthcare provider [3]. OAB can severely affect the quality of life and can impact the social, sexual, occupational and psychological aspects of patient life [2]. Consequently, OAB remains underreported, despite the availability of improved treatments and increased awareness [4,5].

**Figure 1:**
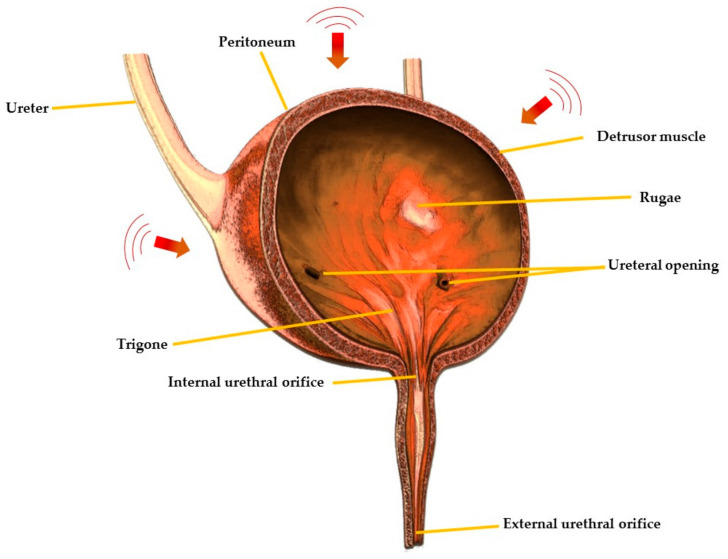
Cross-sectional view of urinary bladder showing different regions.

The etiological factors of OAB can be neurogenic, myogenic and urotheliogenic [6,7]. Abnormal afferent excitability and central sensory processing are the neurogenic causes, and it is prevalent in patients suffering from Parkinson’s disease, multiple sclerosis and cerebrovascular disease [7]. Abnormal transmission of nonadrenergic noncholinergic neurotransmitter is another neurogenic factor that can also cause OAB [8]. The spontaneous contraction of the detrusor muscle (Figure 1) and hypersensitivity to incoming signals is a myogenic factor, whereas changes in ion channel, urothelial signalling and increased afferent activity are urotheliogenic factors [9,10,11]. Changes or disturbances in any of these factors, including any combinations, can cause OAB. Additionally, metabolic derangement, bladder inflammation (interstitial cystitis), and bladder obstruction due to benign prostatic hyperplasia may also cause OAB. These factors typically increase the excitability of the nerve, the detrusor muscle and alter the barrier and sensory functions of the urothelium [6,7]. An urodynamic study reported that 31–68% of OAB patients were diagnosed with bladder outlet obstruction [12]. Yu et al., reported that, in Taiwanese women, hyperlipidaemia is linked with OAB [13]. Moreover, according to a survey conducted on 1359 patients suffering from diabetes mellitus, 22.5% of the patients had OAB syndrome [14]. Chuang et al., also reported that serum levels of CRP, a C-reactive protein which is secreted by the liver in response to inflammation in the body, were higher in patients with OAB compared to those who were not suffering from OAB [15]. However, the causes of OAB differ from one person to another and may involve one or more of the abovementioned factors.

OAB management strategies include non-pharmacological and pharmacological approaches. Non-pharmacological interventions include behavioural and bladder training, whereas pharmacologic therapies include muscarinic receptor antagonists or anti-muscarinic drugs (tolterodine, solifenacin, darifenacin, propiverine, oxybutynin, trospium chloride), α-adrenoceptor antagonists (alfuzosin, doxazosin), β-adrenoceptor antagonists (terbutaline, salbutamol), vitamin D analogues (elocalcitol), a combination of drugs (anti-muscarinic + α-adrenoceptor antagonist), phosphodiesterase inhibitors (sildenafil, taladafil), cyclooxygenase inhibitors (flurbiprofen, indomethacin), voltage-gated calcium channel inhibitor (gabapentin), capsicum plant derived drug, capsaicin (8-methyl-N-vanillyl-6-nonenamide) and the µ-opioid receptor agonist, tramadol [16,17,18,19]. Various drug delivery routes including oral, transdermal, intravesical and vaginal are used for the administration of drugs for the treatment of OAB with the oral route being the most common due to ease of ingestion, increased patient compliance and safety [20]. Currently, anti-muscarinic drugs are the mainstay of oral pharmacotherapy for the treatment of OAB, however, they may be associated with troublesome side effects including constipation and xerostomia [21,22,23,24].

Delivery via the transdermal route is a painless way of systemic drug administration in which the formulation, usually in the form of a gel, cream or patch, is applied onto healthy, intact skin. This route also has many advantages including being suitable for unconscious patients, low incidence of side effects, improved bioavailability due to being able to avoid pre-systemic metabolism, and a large surface area which allows better transdermal absorption of the drug [25,26,27,28]. However, it can cause skin irritation which can lead to potentially serious skin inflammatory problems.

Intravesical therapy involves direct instillation of a drug into the bladder after inserting a catheter into the urethra. Recently, this route has gained popularity in the management of overactive bladder syndrome and is used as an alternative to oral treatment or as a second line in clinical management. However, the urothelium membrane has low permeability which represents a challenge for intravesical drug delivery [29]. Hence, the vaginal route has several advantages such as high permeability to many drugs, avoidance of first-pass hepatic metabolism, and the suitability to accommodate relatively large doses [30]. A delivery system could be used to treat overactive bladder and vaginal dryness simultaneously, which are common issues faced by human females after menopause [31]. However, it is obviously limited to females as well as being associated with local irritation and variability in the extent and rate of drug absorption [32].

Traditional drug delivery systems used in OAB are commonly associated with multiple side effects and low compliance. It is expected that this low compliance may lead to clinical challenges and even therapeutic failure. The aforementioned discussion and challenges warrant the need for a comprehensive review using a systematic search of literature reporting the development and characterisation of formulations for the management of OAB to identify any preferred routes and formulation approaches. Preferred Reporting Items for Systematic Reviews and Meta-Analyses (PRISMA) guidelines [33] were adopted to review the various reported drug delivery strategies and their therapeutic potential.

## 2. Methodology

### 2.1. Search Plot, Information Sources and Screening Process

The search plot based on PRISMA [33] guidelines, which includes identification, screening, eligibility, and inclusion as its key determinants. A systematic search of published research studies from January 2004 to December 2019 was carried out. An inclusive search plot based on Google Scholar, PubMed, MEDLINE, EMBASE and Scopus databases was used. The authors conducted the search using the following search terms: *“overactive bladder syndrome”* OR *“development of OAB formulations”* OR *“drug delivery routes for OAB”* OR *“pharmacological treatment of OAB”* OR *“polymers used for OAB formulations”*. Titles and abstracts of the resultant studies were screened, and studies irrelevant to the scope of the current systematic review were removed. The full texts of the remaining studies were then screened to determine eligibility. Additional studies which were not aligned with the rationale were further removed.

### 2.2. Study Selection

The primary investigators independently evaluated the suitability of eligible studies. The full text was thoroughly screened against the rationale of the systematic review by two reviewers. Any disagreements and differences of opinion among reviewers over the eligibility of a particular study were resolved through a structured discussion.

### 2.3. Data Extraction and Collection

The data was extracted from all the eligible studies using a form (Table S1) developed by the principal investigator, which was used and verified by the lead author. The extracted information was subsequently tabulated using Microsoft Word 2019 following an already published method [34,35,36,37]. The extracted information includes active pharmaceutical ingredient used, drug delivery route, in vitro/in vivo studies, excipients and study characteristics. Moreover, based on the retrieved list of active pharmaceutical ingredients in this systematic search exercise a list of available commercial products was prepared [38].

### 2.4. Risk of Bias Assessment

To assess the risk of bias for all the eligible studies quite a few well established and published checklist and frameworks were explored (Table S2). Interestingly after a detailed and structured exercise, none of them was mapped entirely with our systematic review scope and rationale, although they all were theoretically and epistemologically dense and detailed. To reach a workable solution, we have developed a modified risk of bias assessment with a detailed description of each section based on Cochrane handbook for systematic reviews of interventions and standard quality assessment criteria for evaluating primary research papers from a variety of fields [39,40,41] adopting the mapping and redundancy approach [37,42,43]. The modified framework is reported in Tables S3 and S4 and was thoroughly applied to investigate the risk of bias in each included study. It was assessed in the research rationale, methodology, results, discussion of results and conclusion domains of each study. The authors carried out the initial investigation individually; however, each study’s final recommendation was assigned after the detailed structural panel discussion, and the results are summarised in Table S5.

## 3. Results and Discussion

**Figure 2:**
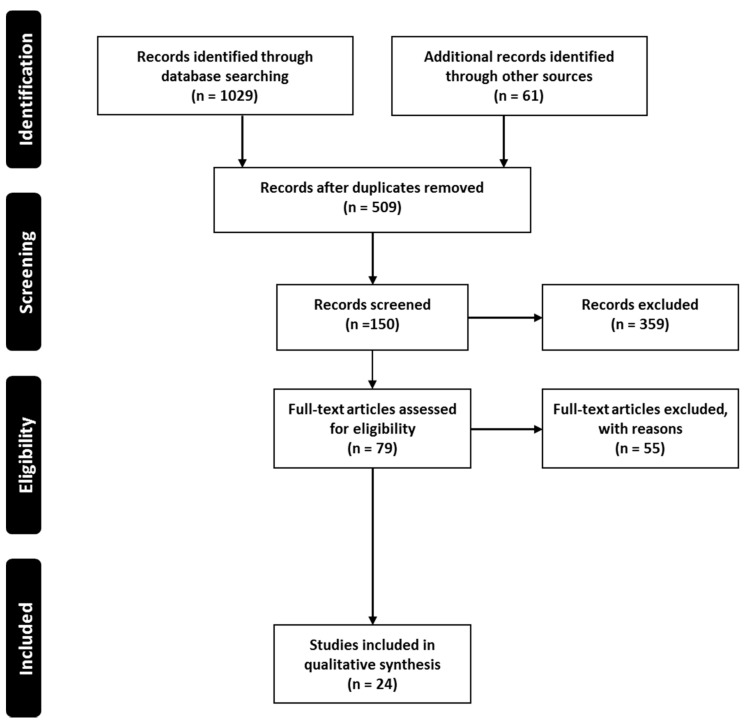
Systematic search and study selection process.

The current systematic search plot yielded 1090 unique studies; however, after removal of duplicates (these were the same articles which appear in search results of multiple databases), 509 studies were included. These studies were further screened by title and abstract, which resulted in the removal of 430 studies. Thus, 79 studies were subjected to full-text screening and finally 24 [44,45,46,47,48,49,50,51,52,53,54,55,56,57,58,59,60,61,62,63,64,65,66,67] were selected and considered eligible to be included in the systematic review for further reporting and analysis (Figure 2). The main reasons for exclusion were a lack of focus on drug delivery and formulation development.

**Figure 3:**
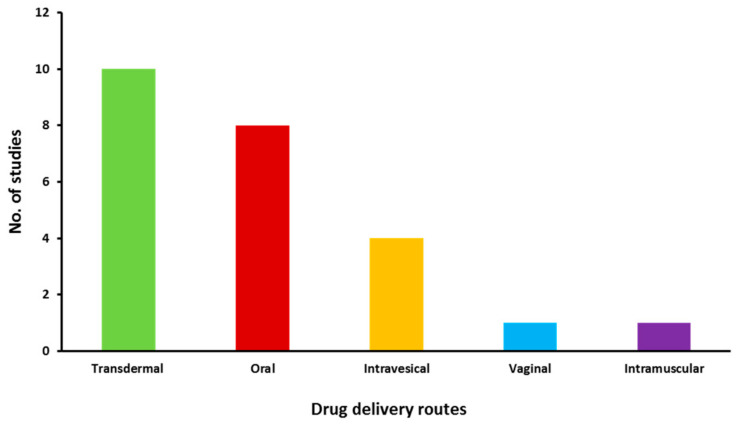
Distribution of eligible studies focused on different drug delivery routes.

**Figure 4:**
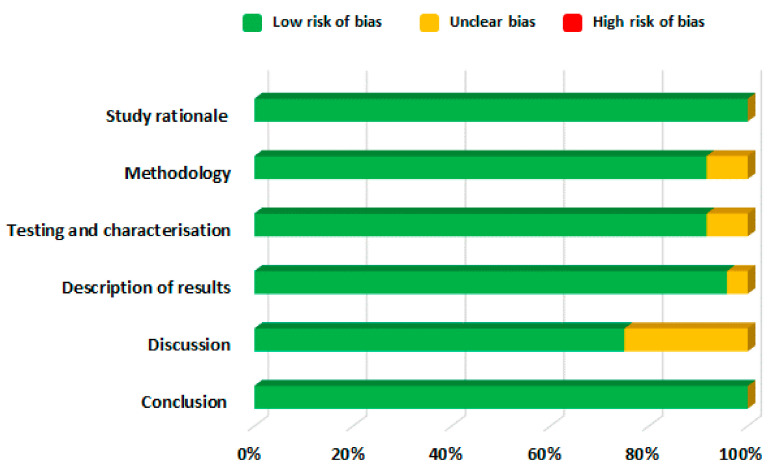
Risk of bias assessment of eligible articles.

The included studies were then further categorised and information was extracted according to the routes of drug delivery and the characteristics described in Table S1, which were then tabulated in Table 1 and discussed separately in succeeding sections. Figure 3 depicts the distribution of the number of studies dedicated to each drug delivery approach. The distribution of risk of bias in the included studies is given in Figure 4. The studies generally had a low risk of bias with 3% unclear bias in the discussion sections, 0.5% in results and 1% in testing and methodology sections. Moreover, information regarding the commercial products of those drugs retrieved in this study was tabulated in Table 2.

### 3.1. Transdermal Route

Transdermal drug delivery systems have proven to be an excellent for OAB patients owing to their painless approach, i.e., direct application of the drug formulation onto healthy and intact skin [25,26]. These systems possess many advantages over other drug delivery routes including non-invasiveness for patients suffering from needle phobia and dysphagia, thus providing a suitable alternative to parenteral and oral routes, as well as minimising the need for multiple administration, hence improving patient compliance [26]. Moreover, in some cases, this route provides enhanced transdermal absorption and improved bioavailability as it avoids pre-systemic metabolism [28].

Oxybutynin is an anti-muscarinic drug and is a selective M1 and M3 receptor antagonist [68]. The presence of both spasmolytic and anticholinergic properties makes it an effective therapeutic option for the treatment of OAB [69]. It is lipophilic and has a short half-life of 1–3 h. Transdermal delivery of oxybutynin is preferred over oral administration for the treatment of OAB as oral administration leads to the production of N-desethyloxybutynin, which is an active metabolite of oxybutynin subject to hepatic first-pass metabolism in the liver and gut and causes severe dryness of the mouth [70,71]. Conversely, transdermal delivery of oxybutynin decreases the onset of the active metabolite, hence reduce the drug induce xerostomia and increases the overall treatment adherence [72]. Various clinical trials have reported that transdermal delivery of oxybutynin is associated with a low incidence of side effects which led researchers to develop transdermal formulations using different polymers and permeation enhancers [73,74]. For example, oxybutynin bioadhesive films were prepared using polyvinyl alcohol (PVA) and sorbitol. The films showed good permeation characteristics across rabbit ear skin, oxybutynin permeation increased linearly for up to 7 h, and 50% of drug permeation was achieved after 24 h [44]. Banu et al., 2010 [45] also developed oxybutynin films containing 2% carbopol-934P and 30% polyethylene glycol which showed 87% drug permeation across rat abdominal skin, whereas the permeation from the formulation containing 2% of ethyl cellulose: carbopol-934P (1:3) and 30% polyethylene glycol was 88%. The results showed that the films were suitable for the transdermal administration of oxybutynin for the treatment of OAB.

**Figure 5:**
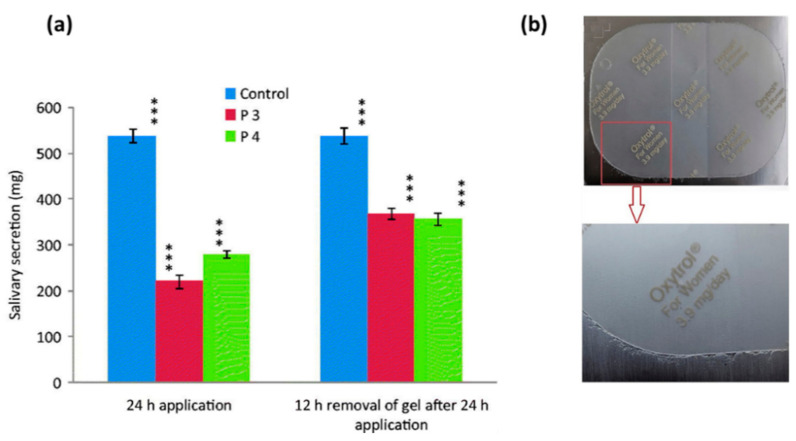
(**a**) Recovery of pilocarpine-induced salivation secretion in rats after transdermal administration of oxybutynin chloride. Statistically significant data (*p* < 0.05 = ***). Reprinted with permission from ref. [47]. Copyright^©^ 2016 Taylor & Francis. (**b**) Appearance of commercial product in primary package. Reprinted with permission from ref. [48]. Copyright 2018 Elsevier.

Although the transdermal route provides many advantages, it is also associated with a few problems, one of which is skin irritation. For example, a topical gel of oxybutynin chloride was approved by the US Food and Drug Administration in 2009 and was intended for application on thighs, buttocks or abdomen daily, but nearly 6% of patients suffered from skin irritation after applying this gel [75]. To overcome this problem, a transdermal spray of oxybutynin was developed using Lutrol F-127 and carbopol-940 as polymers and myristyl lactate and glyceryl monooleate as permeation enhancers. Formulations took 65–70 s to dry and formed a thin film. Permeation studies showed 45–50% drug was permeated and no erythema or oedema was observed, hence it was concluded to be safe to use in managing OAB [46]. Proniosome gel formulations developed by Rajabalaya et al., [47] were also successful in overcoming the skin irritation issue. These contained non-ionic surfactants (Spans (S20, S40, and S60) and Tweens (T20 and T80)), lecithin and cholesterol. Cholesterol provides high permeability and stability, and non-ionic surfactants form a layer that provides deeper penetration of the drug into the skin without causing side effects such as skin irritation. All the formulations had more than 87% entrapment efficiency, which tended to increase with an increase in surfactant concentration. Furthermore, in vitro permeation studies showed that the drug permeation was higher for gels containing Span than gels containing Tween. It was reported that transdermal application of these formulations resulted in decreased pilocarpine-induced salivation as it led to reduced production of N-desethyloxybutynin, N-DEO, (an oxybutynin metabolite) (Figure 5a) and showed highly regenerative surfaces of transitional epithelium. Moreover, commercially available oxybutynin patches may not possess good mechanical properties as pressure-sensitive adhesives are soft and can lead to difficulties removing the patch from the primary packaging (Figure 5b) [48].

To overcome this problem, Wang et al., investigated acrylic adhesives with different functional groups and reported that adhesives with AACONH_2_ functional group displayed good mechanical properties while also enabling the highest in vitro and in vivo (rat model) oxybutynin permeation [48]. Tolterodine is another antimuscarinic drug that is widely prescribed for the treatment of OAB [76]. It is a non-selective muscarinic receptor antagonist, less lipophilic than oxybutynin and does not cross the blood-brain barrier (BBB). Like oxybutynin, oral administration of tolterodine also causes side effects which can lead to patient compliance issues; hence, transdermal delivery of tolterodine would be a suitable alternative [68]. For example, in a study conducted by Pandit et al., [49] a combination of cabopol-934P: hypromellose (1:3) with 30% propylene glycol was effective in producing tolterodine tartrate-based films with high endurance and flexibility and 69% of the drug was permeated through rat skin in in-vitro settings. In a separate study, a transdermal formulation was prepared using carbopol-940 as a gel matrix and N-methyl pyrrolidone as a permeation enhancer. The formulation had a permeation rate of 770 µg cm^−2^ h^−1^ with an absolute bioavailability of 11%. It was reported that the matrix formulation did not cause skin irritation [50]. Liu et al., also used Carbopol-940 and N-methyl pyrrolidone for the development of hydrogels containing 5-hydroxymethyl tolterodine. The formulation resulted in 21% absolute bioavailability, and no skin irritation was observed [51].

**Figure 6:**
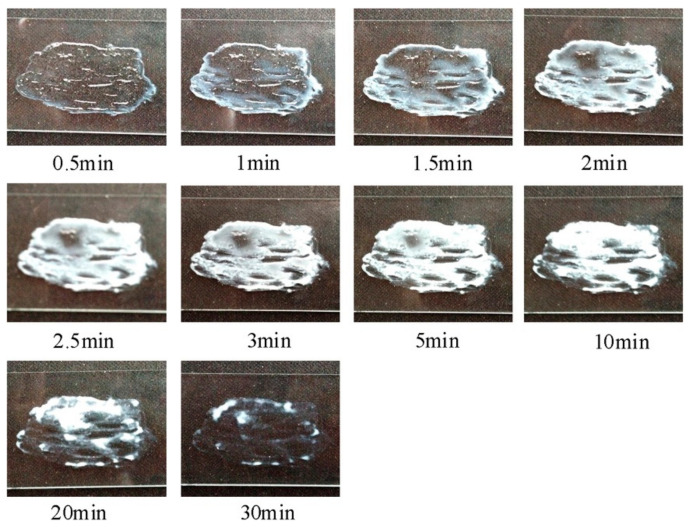
Real-time morphological changes during film-forming process of transparent hydrogels. Reprinted with permission from ref. [52]. Copyright 2014 Elsevier.

In another study, Liu et al., [52] reported the development of a tolterodine hydrogel formulation using Tween 80, hypromellose, Carbopol-980 and hydroxypropyl cellulose (HPC). Morphological changes in the film-forming process of transparent hydrogels are shown in Figure 6. The formulation showed 86% cumulative drug permeation in 24 h. The flux of tolterodine from the formulation was 81.82, 37.15, 18.55 and 15.83 µg cm^−2^ h^−1^, across subcutaneous tissue, dermis, epidermis and full rat skin, respectively. In vivo studies in rats also found that the hydrogel formulation exhibited sustained drug release and permeation over 24 h and higher bioavailability (25%) than tolterodine tablets, with15% bioavailability; hence, making this hydrogel a viable drug delivery system. The applicability of transdermal patches was also assessed where Rajabalaya et al., [53] exploited different grades of Eudragit (E 100, RSPO and RLPO) with various plasticisers and polyvinyl pyrrolidone. These patches had a reduced impact on salivary secretions as compared to oral formulation, thereby proving their usefulness in reduction of common side-effects. The findings of these studies conclude that transdermal films, patches and hydrogels proved to be effective and promising systems for delivery of tolterodine tartrate for the treatment of OAB.

Overall, the findings of these studies confirm that the transdermal delivery of antimuscarinic drugs is useful and provides a suitable alternative to oral administration to avoid side effects.

### 3.2. Intravesical Route

Intravesical therapies have been proposed to achieve inhibition of the overactive detrusor muscle and to avoid high systemic drug levels, thus providing a potentially effective therapeutic option in the management of OAB [77]. They involve the direct instillation of drug into the bladder followed by insertion of a catheter into the urethra [78]. This route of drug administration has gained significant attention in the clinical management of urinary tract disorders including overactive bladder and is considered as a second line treatment after oral pharmacotherapy [79,80]. The urothelium, the lining of urinary bladder, is selective and impermeable due to blood-urine barrier (a high magnitude transurothelial electrical resistance that reflect the net ion flux across the urothelium) [81] which offers many benefits and challenges. The relative impermeability of the urothelium restricts systemic distribution of drugs and may possibly minimises the risk of side effects [82].

Capsaicin, an insoluble vanilloid found in red pepper, is useful in treating overactive bladder and may be delivered via the intravesical route [83]. It is hydrophobic, therefore, capsaicin is prepared in normal saline solution with 30% ethanol for intravesical administration [84] but this vehicle produces submucosal oedema and causes epithelium thinning [85]. To address this, Tyagi et al., [54] encapsulated capsaicin in liposomes and polyethylene glycol-polylactic-co-glycolic acid (PEG-PLGA) polymer was used as a vehicle, maintaining the formulation in a fluid state prior to instillation but transforming into a hydrogel in situ. Three types of capsaicin formulations, liposomes, hydrogel and 30% ethanolic solution, were administered intravesically to rats. The liposomes and ethanolic solution completely blocked micturition reflexes while the hydrogel, although it did not completely block micturition reflexes, led to a significant decrease in bladder contractions. Cystometry showed a significant decrease in calcitonin gene-related peptide (CGRP) staining of afferent nerves in the bladder wall for the liposome and alcoholic solutions, with significant histological changes for those treated with 30% ethanol alone. It was concluded that liposomes are superior vehicles for administration of capsaicin than 30% ethanolic solution. Similarly, liposomes were also employed by Chuang et al., 2009 [55] for delivering botulinum toxin A. Rats treated with lipotoxin ((botulinum toxin A encapsulated in liposomes) exhibited decreased inflammatory reactions with a reduction in SNAP-25 expression and an increase in calcitonin gene-related peptide (CGRP) compared to rats treated with unentrapped botulinum toxin A. Liposomes were found to be an effective vehicle for intravesical delivery of botulinum toxin A avoiding injections and effects on the detrusor muscle.

Although the intravesical drug route has shown promise for the management of OAB, frequent insertion of a catheter may lead to poor patient acceptability and compliance. Hopmann et al., in 2015 [56] fabricated an implant comprising drug in poly(D,L-lactide-co-glycolide)-co-polyethylene glycol di-block copolymer microspheres embedded into a polydimethylsiloxane absorbable foam matrix. The device was shown to extend the release for up to four weeks in artificial urine and has the potential to extend the delivery of the highly soluble, trospium chloride. Entrapment in a lipid base may also control the release of this drug [57]. Three systems comprising glyceryl tristearate, mini tablets made using compression, extrudates formed by solid-lipid extrusion and mini-moulds manufactured via a melting and casting technique. The preparation method impacted the drug release kinetics with extrudates and mini-tablets extending release of trospium chloride for more than 5 days; however, the drug release from the mini-mould was better. Overall, it was concluded that lipids may be a suitable matrix for controlling the release of highly soluble drugs and preparing delivery systems of a small size making insertion and excretion achievable, thus addressing some of the issues with this delivery route.

The above-mentioned studies show that intravesical route is an effective and suitable approach for the administration of drugs in the treatment of OAB with less incidence of side effects as compared to oral pharmacotherapy.

### 3.3. Vaginal Route

The vaginal route is hugely significant for drug delivery in women suffering from OAB. It has established merits compared with other routes enabling extended drug release and action. Also, avoidance of first pass metabolism may reduce dosing frequency which may improve patient compliance. The delivery of drugs via this route is considered favourable in managing overactive bladder and vaginal dryness simultaneously, which are common issues faced by human females after menopause [86,87,88,89,90,91,92].

**Figure 7:**
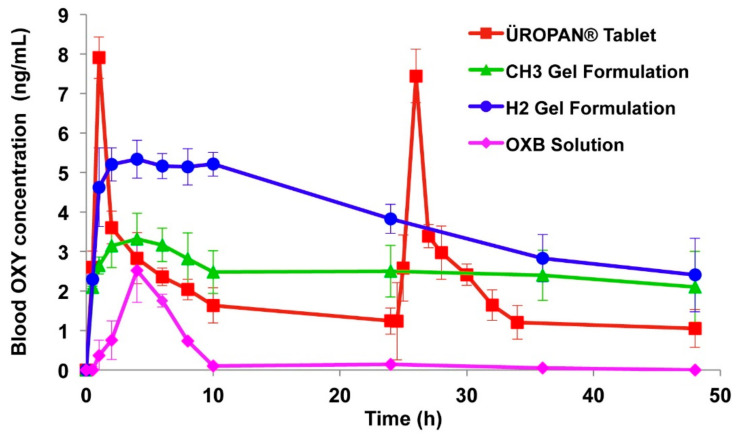
Blood oxybutynin concentration profiles of oral and vaginally administered formulations, CH3 (1% *w*/*v* glacial acetic acid and 3% *w*/*v* Chitosan H), H2 (2% *w*/*v* hypromellose K100M), Reprinted with permission from ref. [58]. Copyright 2013 Elsevier.

Bioadhesive gels are the most commonly used therapeutic delivery systems to prolong the residence time in the vagina. Mucoadhesive gels of oxybutynin were developed using polymers such as Poloxamer 407, hypromellose K100M and chitosan. The gel formulation containing hypromellose K100M (2% *w*/*v*, CH2) exhibited better mucoadhesion, adhesiveness, cohesiveness and viscosity than chitosan and poloxamer gel formulations. Permeation studies across vaginal mucosa also confirmed better permeation of oxybutynin from hypromellose K100M gel formulation. In vivo studies in rabbits showed that hypromellose K100M gels (2% *w*/*v*, CH2) resulted in the highest relative bioavailability (Figure 7). Conclusions were that oxybutynin mucoadhesive vaginal gels are innovative and a promising drug delivery system that can be used safely for vaginal dryness and overactive bladder after menopause [58].

### 3.4. Intramuscular Route

Intramuscular route is another suitable alternative for the management of OAB. This route is suitable for unconscious patients or for drugs where pharmacokinetic findings recommend avoiding other routes of administration [93]. Microsphere based intramuscular formulations are designed to maintain drug release over a sustained period of time, reduce dose related adverse effects and improve therapeutic potential [94,95,96]. Sun et al., 2010 [59] developed intramuscular depot formulations of tolterodine using PLGA microspheres as a carrier r. PLGA concentration affected the encapsulation efficiency and led to an increase from 63 to 79% when polymer concentration was increased from 180 to 230 mg/mL. Drug entrapment efficiency also increased upon adding palmitic or stearic acid. The bioavailability of these formulations was studied in beagle dogs and a sustained drug release was observed for ~18 h; however, an initial burst release was also evident. The results concluded that these formulations resulted in continuous inhibition of muscarinic receptors in comparison to oral formulations that inhibit muscarinic receptor in a pulsatile fashion and can provide a more effective treatment for OAB patients.

### 3.5. Oral Route

**Figure 8:**
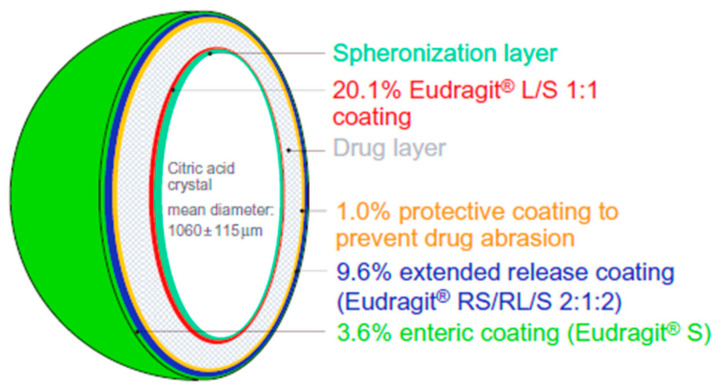
Illustration depicted the formulation principle of membrane-coated multiple units extended release pellets. Reprinted with permission from ref [60]. Copyright 2009 Taylor & Francis.

Oral pharmacotherapy is the mainstay of the treatment of OAB, especially for antimuscarinic drugs [77]. It is the most convenient and preferred route of drug administration due to ease of production, high patient compliance, cost-effectiveness and flexibility in dosage form [88]. However, the oral delivery of antimuscarinic drugs may lead to some side effects including constipation and xerostomia [21,22,23,24]. Extended release oral formulations are of enormous importance and provide great benefits. Propiverine is used for the treatment of OAB via the oral route and its solubility is pH dependent which presents problems in the development of extended release formulations. Different coatings levels of Eudragit polymer and propiverine were applied to the citric acid crystals in a sequential manner to prepare extended release pellets (Figure 8). Drug release profiles from the developed pellet formulations in the presence and absence of pH modifier and the free-base and hydrochloride salt were comparatively evaluated. The main objective of this formulation design was to create a favourable environment for drug dissolution within the pellet while extending the drug release. Upon contact with the dissolution medium, osmotic pressure built up inside the pellet core, which in turn caused drug release through the polymer coating. The results showed that, with higher level of coating, the drug release and citric acid release was reduced. In fact, citric acid release was slower than the drug release and stayed within the pellets for more than 16 h. However, if microcrystalline cellulose pellets are employed as a starting core then the drug release is pH-dependant. Moreover, noticeable differences were noticed between the formulations containing the free drug base and those with the hydrochloride salt as a result of an altered microenvironmental pH. It was concluded that this approach is effective and feasible for extended release formulations of propiverine for managing OAB [60].

Pradhan et al., [61] developed coated tablets of tolterodine tartrate using hypromellose 2208 and 2910 that extended release for 10 h in vitro and was similar to a marketed product. Similar results were obtained from in vivo studies where the pharmacokinetic parameters of the developed formulation and commercial formulation were not significantly different from each other with formulations being bioequivalent. Therefore, the hypromellose based matrix tablet could be a suitable alternative to sustained release capsules. Patil et al., [62] also developed extended release pellets of tolterodine tartrate using mannitol as an osmotic agent along with hypromellose. These pellets were then filled into capsules of suitable sizes and the in-vitro drug release compared with Detrol LA^®^; a generic version of the tolterodine tartrate. It was concluded that the extended release capsule administered once a day can achieve similar results as that of Detrol LA^®^. Similarly, extended release matrix tablets of oxybutynin chloride were prepared using combinations of polymers including hypromellose K4M, K100M, Carbopol, ethyl cellulose, PVP, and sodium alginate. A formulation containing hypromellose K4M along with ethyl cellulose demonstrated controlled drug release in buffer for 24 h [63]. This shows that extended release formulations are suitable for the treatment of OAB as they are required to be administered only once in a day which reduces the need for multiple administration of doses as compared to immediate release formulations. Sudarsan et al., [64] developed darifenacin hydrobromide reservoir tablets using ethyl cellulose as a coating agent. These formulations were compared with the marketed product Enablex^®^. Tablets showed good friability and 90% drug release over 12 h in 0.1 M HCl, similar to Enablex^®^ tablets. SreeHarsha et al., [65] developed a self-emulsifying drug delivery system (SEDDS) for the poorly soluble darifenacin using surfactant (Labrafil 1944 CS) and co-surfactant (polyethylene glycol 400) in a ratio of 2:1 with peanut oil. The dissolution rate of the developed formulations was greater than pure darifenacin in in vitro dissolution studies.

**Figure 9:**
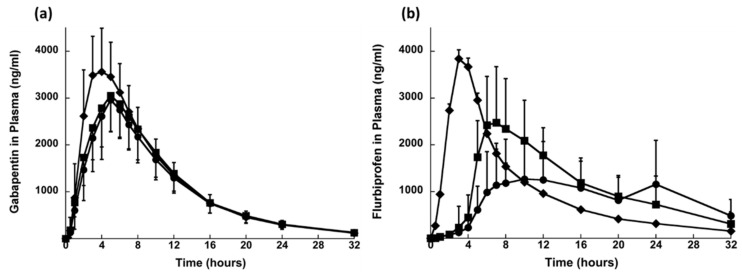
Plasma profiles of (**a**) gabapentin and (**b**) flurbiprofen after single dose administration in fed conditions of (●) TLT_hypromellose Type I, (■) TLT_hypromellose Type II and (♦) Immediate release dosage form (mean values ± SD; *n* = 24). Reprinted with permission from ref. [66]. Copyright 2017 Elsevier.

In 2008, a patent has been filed disclosing the synergistic effect gabapentin and flurbiprofen in relieving the OAB symptoms [96]. Based on this patent’s findings Sonvico et al., 2017 [66] has developed an oblong shaped tri-layered tablet with multi drug release kinetic profiles containing gabapentin and flurbiprofen. Layer A (the top layer) and B (the middle layer) contained gabapentin for prolonged and immediate release, respectively. Layer C (the bottom layer) contained flurbiprofen for delayed but prolonged release. During in vitro dissolution testing, layer B disintegrated within a few minutes leading to the eventual splitting of layer A and C. Layer A started to float and layer C sank down in the bottom. Layer A floated for about 7 h and for layer C, there was no flurbiprofen release in the first 60 min. After transferring the layer C to pH 7.2 medium, accelerated dissolution was observed. The in vitro drug release assessment confirmed the programmed drug delivery aspects of this tri-layer fixed dose formulation. Additionally, a higher bioavailability of gabapentin was noticed when delivered in fed condition (30 min after the meal) to human volunteers in comparison to dose administration 10 min before meal or in fasting condition, Figure 9. The researchers of this particular study have argued that these findings are supporting of the gastroretention potential of gabapentin prolonged release layer (layer A). The two drugs were delivered at different anatomical sites, since the food presence prolonged the gastric absorption of gabapentin from the floating layer and delayed the flurbiprofen absorption. Moreover, the delayed or intestinal specific release of flurbiprofen was realised using a matrix-based polymer combination system negating the necessity of film coating. The results show that tri-layered tablet formulation provided modified release of drugs which may be a suitable option for managing OAB [66].

Additionally, the fast dissolving drug delivery systems have advantages for geriatric and paediatric patients due to rapid disintegration, ease of administration or self-administration, and non-requirement for chewing or water. Fast dissolving films of darifenacin hydrobromide were developed by Abbas et al., [67] using PVA, Tween 80 and glycerol. Different types and concentrations of superdisintegrants including sodium starch glycolate, croscarmellose sodium and Indion 414 were investigated. The results revealed that the formulation containing 4% *w*/*w* indion 414, 30% *w*/*w* glycerol, 2% *w*/*v* PVA, 0.5% *w*/*v* tween 80 and 7.5 mg of darifenacin hydrobromide was an optimum formulation by showing the shortest disintegration time of 31 Sec. Such formulations provide faster therapeutic effects to patients suffering from OAB.

Overall, for oral drug delivery route various dosage form designs were identified which are easy to develop, such as reservoir drug delivery systems, tri-layered tablets, coated tablets and loading of microspheres in capsules.

## 4. Conclusions

The current systematic review has identified the various formulations strategies, marketed products and has aided the comparison of diverse formulations approaches intended for different drug delivery routes where each route has its importance and challenges. The exploitation of different drug delivery routes can improve patient compliance and adherence, and advances in the formulation of dosage forms can help patients to get effective treatment with minimal side effects. This systematic review found evidence regarding the frequent use of the transdermal route which has led to commercial products; however, other routes such as oral, intravesical, vaginal and intramuscular were also identified. It can be concluded from the current systematic review that drug delivery routes other than oral are used to avoid the side effects caused by oral administration of drugs. For example, the oral delivery of antimuscarinic drugs can potentially cause bothersome side effects. Moreover, this review has identified the need for robust in vitro tests which should be aligned with in vivo/clinical testing. Generally, it is expected that the detailed discussion of various studies and enlisting of their marketed products will be a valuable resource for pharmaceutical scientists to better understand and comprehend the existing literature and challenges, which will be anticipated to provide a basis for designing and fabricating new effective formulations to manage OAB.

## 5. Limitations of Current Systematic Review

The current study is providing the commercial product information only to showcase what kind of different dosage forms are available in the market. Authors are not claiming that the list is showing all the OAB commercial products available in the market.

## Figures and Tables

**Table 1:** Summarised characteristics of eligible studies.

Study ID	Drug	Drug Delivery Route	Type of Investigation	Excipients	Study Characteristics	Reference
Nicoli et al., 2006	Oxybutynin	Transdermal	In vitro	PVA, sorbitol	The developed oxybutynin films showed good permeation characteristics across rabbit skin, oxybutynin permeation increased in a linear way for up to 7 h and 50% of the drug permeation was achieved after 24 h.	[44]
Banu et al., 2010	Oxybutynin	Transdermal	In vitro	Ethyl cellulose, Carbopol-934P and PG	Films containing 2% Carbopol-934P and 30% PG showed 87.28% drug release across rat abdominal skin whereas drug release from formulation containing 2% of ethyl cellulose: Carbopol-934P (1:3) and 30% PG was 88.32%.	[45]
Bakshi et al., 2008	Oxybutynin	Transdermal	In vitro	Lutrol F-127, Carbopol-940, myristyl lactate and glyceryl monooleate	The droplet size delivered from the formulations was within the range of 5–50 µm. The drug dose delivered per actuation of the pump was within the range of 101–106% by each formulation. Permeation studies were carried out on rabbit ear skin and showed that drug release was within the range of 45–50% during the period of 24 h.	[46]
Rajabalaya et al., 2016	Oxybutynin chloride	Transdermal	In vitro and in vivo *(rat model)*	Span (S20, S40, and S60) and Tween (T20 and T80), cholesterol and glycerol distearate, isopropyl alcohol	All the formulations showed more than 87% entrapment efficiency which tended to increase with increase in surfactant. In vitro permeation studies determined that percent cumulative permeation after 8 h was higher for gels containing Span than gels containing Tween.	[47]
Wang et al., 2018	Oxybutynin	Transdermal	In vitro and in vivo *(rat model)*	Acrylic adhesives, span 80	The patch with AACONH_2_ functional group acrylic adhesive had the highest (763.5 ± 58.8 μg/cm^2^) oxybutynin cumulative levels during in vitro skin permeation study. The relative bioavailability of the developed patches was 97% in rats.	[48]
Pandit et al., 2009	Tolterodine tartrate	Transdermal	In vitro	Ethyl cellulose, Carbopol-934P, Hypromellose and PG	Different concentrations of polymers and PG were investigated. Combination of cabopol-934P: hypromellose (1:3) with 30% PG was effective in producing films of high endurance flexibility and with uniform drug content. Permeation study showed that there was 68.72% drug release across rat abdominal skin and 81.12% release across cellophane membrane for 12 h.	[49]
Sun et al., 2013	Tolterodine	Transdermal	In vitro and in vivo *(rabbit model)*	Carbopol 980, N-methyl pyrrolidone	The formulation showed permeation rate of 770.19 µg cm^−2^ h^−1^ during in vitro percutaneous permeation experiment. The absolute bioavailability was 11.47% during pharmacokinetic studies.	[50]
W. Liu et al., 2017	5-hydroxymethyl tolterodine	Transdermal	In vitro and in vivo *(rat model)*	Carbopol 934, 940 and 980,	The formulation showed 20.7% absolute bioavailability during in vivo studies and no skin irritation was observed during skin irritation study.	[51]
X. Liu et al., 2014	Tolterodine	Transdermal	In vitro and in vivo *(rat model)*	Tween 80, Hypromellose, HPC, Carbopol 980	The formulation showed 86.02% cumulative drug release rate in 24 h. The flux of tolterodine from the formulation was 81.82, 37.15, 18.55 and 15.83 µg cm^−2^ h^−1^, across subcutaneous tissue, dermis, epidermis and full rat skin, respectively.	[52]
Rajabalaya et al., 2017	Tolterodine tartrate	Transdermal	In vitro and in vivo *(rat model)*	Eudragit (E 100, RSPO and RLPO), dibutyl sebacate (DBS), dibutyl phthalate (DBP) and triethyl citrate (TEC) and polyvinyl pyrrolidone (PVP)	Drug loaded formulations (i) E100, PVP, and DBS and (ii) RSPO, RLPO, PVP and DBS showed highest percent cumulative permeation and permeation rate during in vitro permeation studies	[53]
Tyagi et al., 2004	Capsaicin	Intravesical	-	Phosphatidylcholine, Cholesterol, PEG-PLGA-PEG, Saline solution with 30% ethanol	Three types of formulations, liposomes, hydrogel and 30% ethanolic solution, were developed. The results showed that the liposomes and 30% ethanolic solution completely blocked the micturition reflexes after intravesical administration. However, hydrogel of capsaicin was not successful in blocking the micturition reflexes completely but there was significant decrease in bladder contractions	[54]
Chuang et al., 2009	Botulinum toxin A	Intravesical	In vivo *(rat model)*	l-α-phosphatidylcholine and cholesterol	Intravesical delivery of lipotoxin (botulinum toxin A encapsulated in liposomes) was investigated to evaluate the effect of lipotoxin on bladder hyperactivity. The results showed that intercontraction interval was 21.1%.	[55]
Hopmann et al., 2015	Barium sulphate	Intravesical	In vitro	PLGA-PEG, polydimethylsiloxane	Samples were incubated in artificial urine for 27 days and CESP parameters including temperature, pressure and pressure release gradient were measured. The spheres and pills at constant temperature of 65 °C and pressure of 50 bar were totally dissolved in 27 days with pressure release gradient of 5 and 20 bar/min, respectively.	[56]
Haupt et al., 2013	Trospium chloride	Intravesical	In vitro	Glyceryl tristearate, magnesium stearate	Extrudates and mini-tablets released drug for more than 5 days. The drug release from mini-moulds was very low or negligible and it was concluded that lipids provides good matrix for highly soluble drugs and with drug loading of only 30% the drug was released from several days up to weeks.	[57]
Tuğcu-Demiröz et al., 2013	Oxybutynin	Vaginal	In vitro and in vivo *(rabbit model)*	Chitosan, Hypromellose (K100M) and Poloxamer 407 (Pluronic F 127).	The hypromellose K100M formulation resulted in suitable permeation characteristics across the vaginal mucosa and also resulted in highest relative bioavailability and AUC during in vivo studies.	[58]
Sun et al., 2010	Tolterodine	Intramuscular	In vivo *(rat model)*	PLGA, palmitic acid, stearic acid	Drug entrapment efficiency was increased upon adding palmitic or stearic acid. The formulation was administered intramuscularly to beagle s. A sustained release following an initial burst was observed for 18 days.	[59]
Ploen et al., 2009	Propiverine hydrochloride	Oral	In vitro	Citric acid, Eudragit	At higher coating levels e drug release and citric acid release was reduced. The pellets extended the drug release for more than 16 h.	[60]
Pradhan et al., 2014	Tolterodine-l-tartrate	Oral	In vitro and in vivo *(human volunteers)*	Hypromellose 2208 and hypromellose 2910	The formulation showed sustained release profile up to 10 h during in vitro dissolution testing. Similar results were obtained from in vivo results.	[61]
Patil et al., 2017	Tolterodine tartrate	Oral	In vitro	Mannitol, hypromellose	The in vitro dissolution profile of extended release capsule was similar to Detrol LA and resulted in more than 85% release during the period of 12 h.	[62]
Naik et al., 2016	Oxybutynin chloride	Oral	In vitro	Hypromellose K4M, K100M, Carbopol, ethyl cellulose, PVP, sodium alginate	The formulation containing Hypromellose K4M along with ethyl cellulose was an optimised formulation that showed controlled drug release for period of 24 h and resulted in cumulative release of 95.59% of drug release. The formulation followed first order kinetics.	[63]
Sudarsan et al., 2014	Darifenacin hydrobromide	Oral	In vitro	Ethyl cellulose, povidone, magnesium stearate	Formulation with highest level of ethyl cellulose coating was an optimised formulation as the results were satisfactory with regards to all parameters and drug release profile was similar to the marketed product.	[64]
SreeHarsha et al., 2019	Darifenacin	Oral	In vitro	Surfactant (Labrafil 1944 CS) and co-surfactant (polyethylene glycol 400) and peanut oil	SEDDS were developed using surfactant, co-surfactant and peanut oil. The average globule size of SEDDS was less than 200 nm and depicted negative zeta potential. The rate of dissolution of the developed formulations was also increased upon comparison with pure darifenacin.	[65]
Sonvico et al., 2017	Gabapentin and flurbiprofen	Oral	In vitro and in vivo *(human volunteers)*	Hypromellose K15M, PEO, PVP K30, Mannitol, sodium croscarmellose, sodium alginate, B-cyclodextrin	This was a tri-layered formulation and, during in vitro dissolution testing, layer B disintegrated in few minutes splitting the layer A and C eventually. Layer A started to float and layer B sank down in the bottom. Layer A floated for about 7 h and for layer C there was no flurbiprofen release in the first 60 min. After transferring the layer C to pH 7.2 medium, accelerated dissolution was observed	[66]
Abbas et al., 2019	Darifenacin hydrobromide	Buccal	In vitro	PVA, Tween 80, croscarmellose sodium, sodium starch glycolate, indion 414	The formulation containing 4% *w*/*w* indion 414, 30% *w*/*w* glycerol, 2% *w*/*v* PVA, 0.5% *w*/*v* tween 80 and 7.5 mg of darifenacin hydrobromide was an optimum formulation by showing shortest disintegration time of 31.28 s.	[67]

**Table 2:** List of commercial products of drugs used for managing OAB [38].

Drug	Formulation Type	Company Name	Marketed Name
Oxybutynin hydrochloride	Tablet	Alliance Healthcare Ltd.	Oxybutynin 2.5, 3 and 5 mg tablets
	Modified-release tablet	Janssen-Cilag Ltd.	Lyrinel XL
	Oral solution	Brillpharma Ltd.	Oxybutynin 2.5 mg/5 mL and 5 mg/5 mL oral solution
	Transdermal patch	Orion Pharma (UK) Ltd.	Kentera 3.9 mg/24 h patches
Tolterodine tartrate	Tablet	Sandoz Ltd.	Tolterodine 1 mg tablets
	Modified-release capsule	Aspire Pharma Ltd.	Neditol XL
Capsaicin	Cream	Teva UK Ltd.	Zacin 0.025% cream
			Axsain 0.075% cream
	Cutaneous patch	Grunenthal Ltd.	Qutenza 179 mg
Botulinum toxin type A	Powder for solution for injection	Allergan Ltd.	Botox 50, 100 and 200 unit powder for solution for injection vials
		Galderma (UK) Ltd.	Azzalure 125 unit powder for solution for injection vials
		Ipsen Ltd.	Dysport 300 and 500 unit powder for solution for injection vials
Trospium chloride	Tablet	Galen Ltd.	Flotros 20 mg tablets
	Modified-release capsule	Contura Ltd.	Regurin XL 60 mg capsules
Darifenacin hydrobromide	Modified-release tablet	Norgine Pharmaceuticals Ltd.	Emselex 7.5 and 15 mg modified-release tablets
Propiverine hydrochloride	Tablet	Advanz Pharma	Detrunorm 15 mg tablets
	Modified-release capsule	Advanz Pharma	Detrunorm XL 30 and 45 mg capsules
Gabapentin	Tablet	A A H Pharmaceuticals Ltd.	Gabapentin 600 mg tablets
	Capsule	Accord Healthcare Ltd.	Gabapentin 100, 300 and 400 mg capsules
	Oral solution	A A H Pharmaceuticals Ltd.	Gabapentin 50 mg/mL oral solution sugar free
		Imported (United States)	Neurontin 250 mg/5 mL oral solution
Flurbiprofen	Tablet	Mylan	Flurbiprofen 50 and 100 mg tablets
	Lozenge	Reckitt Benckiser Healthcare (UK) Ltd.	Strefen Honey and Lemon 8.75 mg lozenges

## Supplementary Materials

The following are available online at https://www.mdpi.com/article/10.3390/ph14050409/s1, Table S1: Form used for the data extraction from eligible studies, Table S2: List of various quality assessment and risk of bias assessment tools considered, Table S3: Detailed description of risk of bias assessment process, Table S4: Summary of an approach for assessing the risk of bias within and across studies, Table S5: Risk of bias findings of all the eligible studies.
