# Supplementary material for: Drug Delivery Approaches for Managing Overactive Bladder (OAB): A Systematic Review
Source: Pharmaceuticals (Basel). 2021 Apr 26;14(5):409. doi: 10.3390/ph14050409 (PMC8146593; doi:10.3390/ph14050409)
Supplement: Supplementary file 1 [file pharmaceuticals-14-00409-s001.zip › pharmaceuticals-1130491-supplementary.pdf]

Supplementary

Drug Delivery Approaches for Managing Overactive Bladder (OAB): A Systematic Review

Zara Khizer [1], Amina Sadia [2], Raman Sharma [3], Samia Farhaj [1], Jorabar Singh Nirwan [1], Pratibha Kakadia [4], Talib Hussain [5], Abid Mehmood Yousaf [5], Yasser Shahzad [5], Barbara R. Conway [1] and Muhammad Usman Ghori [1,*]

[1] Department of Pharmacy, School of Applied Sciences, University of Huddersfield, Huddersfield, UK HD1 3DH.
[2] District Headquarter Hospital, Sahiwal, Pakistan
[3] Parkside Medical Practice, Horton Park Health Centre, Bradford, UK BD7 3EG
[4] Behavioral & Social Science, Integrative Behavioral Health Research Institute, California, USA
[5] Department of Pharmacy, COMSATS University Islamabad, Lahore, Pakist

Table S1: Form used for the data extraction from eligible studies

Study parameters	Description
Study ID	
Active pharmaceutical ingredient (API)	
Drug delivery route	
Investigation type (*in-vitro* or *in-vivo, animal model*)	
Used excipients	
Study characteristics	
Reference	

Table S2: List of various quality assessment and risk of bias assessment tools considered

Number	Risk of bias/quality assessment frameworks
1	Academy of Nutrition & Dietetics/American Dietetic Association Quality Criteria Checklist
2	Critical Skills Appraisal Program (CASP) Checklists
3	Clinical Trials Assessment Measure
4	Cochrane Back Review Group Methods 2009
5	Cochrane Effective Practice and Organisation of Care (EPOC) Tools
6	Cochrane Handbook
7	Cochrane Risk of Bias Tool
8	Cochrane Risk of Bias Tool 2.0
9	Downs and Black
10	GRADE approach
11	Jadad Scale
12	Joanna Briggs Institute Critical Appraisal Tools
13	Kratochwill et al. (2010) recommended criteria
14	Mixed Methods Appraisal Tool (MMAT)
15	Modified Coleman Methodology Scores
16	National Health and Medical Research Council (Australian Government) Scale
17	NICE Checklists (National Institute for Health and Care Excellence)
18	National Heart, Lung, and Blood Institute (NHLBI) Quality Assessment Tools
19	OSTEBA statements (Basque Office for Health Technology Assessment)
20	Oxford Center for Evidence-based Medicine Levels of Evidence (CEBM)
21	PEDro (Physiotherapy Evidence Database) Scale
22	Rosendal scale
23	SIGN (Scottish Intercollegiate Guidelines Network) Checklist for Cohort Studies
24	Standard quality assessment criteria for evaluating primary research papers from a variety of fields
25	STROBE (Strengthening The Reporting of Observational Studies in Epidemiology)
26	Tool for the assessment of Study quality and reporting in Exercise (TESTEX)

Table S3: Detailed description of risk of bias assessment process.

Study area	Description	Reviewer's judgement
**Research rationale**	Explanation of research rationale including aim and objectives, allowing sufficient knowledge to facilitate the decision making process.	Was research rationale (hypothesis and/or aim and/or objectives) adequately described?
**Description of methodology**	Methodology of the experiments are described adequately allowing sufficient knowledge to facilitate the decision making process and there are probable chances of replication.	Was research methodology adequately/sufficiently described?
**Characterisation and testing**	Characterisation and testing techniques are relevant and described in sufficient detail that there are probable chances of replication.	Were research characterisation and testing techniques adequately/sufficiently described?
**Description of results**	Results are described in sufficient detail, allowing sufficient knowledge to facilitate the decision making process.	Were results adequately/sufficiently described?
**Description of discussion**	Results are plausibly discussed, allowing sufficient knowledge to facilitate the decision making process.	Were results discussed adequately/sufficiently described?
**Description of conclusions**	Conclusions are described in accordance with set hypothesis, allowing sufficient knowledge to facilitate the decision making process.	Were conclusions adequately/sufficiently described and relevant to rationale?
**Other source of bias**	Any other important concerns not addressed on the described key areas of the present tool.	Was study free of any unexpected problem?

Table S4: Summary of an approach for assessing the risk of bias within and across studies

Risk of bias	Sign	Interpretation	Within study	Across study
Low	+	Probable bias evidence implausible to seriously affect the findings with no impact on confidence	All major areas have low risk of bias	Most of the studies are found to have low risk of bias, not impact the result interpretation process.
Unclear	?	Probable bias evidence to raise doubts regarding findings with mild to moderate impact on confidence	One or more areas of the study has unclear risk of bias.	Most of the studies are found to have low/or unclear risk of bias, not impact the result interpretation process.
High	-	Probable bias evidence seriously affecting the finings with high impact on confidence	One or more areas of the study has high risk of bias.	High proportion of studies have high risk of bias, sufficiently impacting the result interpretation process.

Table S5: Risk of bias findings of all the eligible studies

Study ID	Risk of bias parametric domains					
	Research rationale	Description of methods	Characterisation and testing	Description of results	Discussion	Overall conclusions
Nicoli et al., 2006	+	+	+	+	+	+
Banu et al., 2010	+	+	+	+	+	+
Bakshi et al., 2008	+	+	+	+	+	+
Rajabalaya et al., 2016	+	+	+	+	+	+
Wang et al., 2018	+	+	+	+	+	+
Pandit et al., 2009	+	?	+	+	+	+
Sun et al., 2013	+	+	+	+	+	+
W. Liu et al., 2017	+	+	+	+	+	+
X. Liu et al., 2014	+	+	+	+	?	+
Rajabalaya et al., 2017	+	+	+	+	+	+
Tyagi et al., 2004	+	+	+	+	?	+
Chuang et al., 2009	+	+	?	+	+	+
Hopmann et al., 2015	+	+	+	+	?	+

Haupt et al., 2013	+	+	+	+	?	+
Tuğcu-Demiröz et al., 2013	+	+	+	+	+	+
Sun et al., 2010	+	+	+	?	+	+
Ploen et al., 2009	+	+	+	+	+	+
Pradhan et al., 2014	+	+	+	+	+	+
Patil et al., 2017	+	+	+	+	+	+
Naik et al., 2016	+	?	+	+	?	+
Sudarsan et al. 2014	+	+	+	+	?	+
SreeHarsha et al., 2019	+	+	+	+	+	+
Sonvico et al., 2017	+	+	+	+	+	+
Abbas et al., 2019	+	+	?	+	+	+

+ Low risk of bias ? Unclear bias - High risk of bias
